# Combining GWAS and comparative genomics to fine map candidate genes for days to flowering in mung bean

**DOI:** 10.1186/s12864-024-10156-x

**Published:** 2024-03-12

**Authors:** Kevin O. Chiteri, Ashlyn Rairdin, Kulbir Sandhu, Sven Redsun, Andrew Farmer, Jamie A. O’Rourke, Steven B. Cannon, Arti Singh

**Affiliations:** 1 Department of Agronomy, Iowa State University, Ames, IA United States; 2 Agriculture and Agri-Food, Morden, MB Canada; 3 National Center for Genome Resources, Santa Fe, NM 87505 United States; 4 USDA - Agricultural Research Service, Corn Insects, and Crop Genetics Research Unit, Ames, IA United States

**Keywords:** Mung bean, Pulses, Legumes, Genomics, Marker-assisted breeding, Orthologs, Homologs

## Abstract

**Background:**

Mung bean (*Vigna radiata* (L.) Wilczek), is an important pulse crop in the global south. Early flowering and maturation are advantageous traits for adaptation to northern and southern latitudes. This study investigates the genetic basis of the Days-to-Flowering trait (DTF) in mung bean, combining genome-wide association studies (GWAS) in mung bean and comparisons with orthologous genes involved with control of DTF responses in soybean (*Glycine max* (L) Merr) and Arabidopsis (*Arabidopsis thaliana*).

**Results:**

The most significant associations for DTF were on mung bean chromosomes 1, 2, and 4. Only the SNPs on chromosomes 1 and 4 were heavily investigated using downstream analysis. The chromosome 1 DTF association is tightly linked with a cluster of locally duplicated *FERONIA* (*FER*) receptor-like protein kinase genes, and the SNP occurs within one of the *FERONIA* genes. In Arabidopsis, an orthologous *FERONIA* gene (*AT3G51550*), has been reported to regulate the expression of the *FLOWERING LOCUS C* (*FLC*). For the chromosome 4 DTF locus, the strongest candidates are *Vradi04g00002773* and *Vradi04g00002778*, orthologous to the Arabidopsis *PhyA* and *PIF3* genes, encoding phytochrome A (a photoreceptor protein sensitive to red to far-red light) and phytochrome-interacting factor 3, respectively. The soybean *PhyA* orthologs include the classical loci *E3* and *E4* (genes *GmPhyA3, Glyma.19G224200,* and *GmPhyA2, Glyma.20G090000*). The mung bean *PhyA* ortholog has been previously reported as a candidate for DTF in studies conducted in South Korea.

**Conclusion:**

The top two identified SNPs accounted for a significant proportion (~ 65%) of the phenotypic variability in mung bean DTF by the six significant SNPs (39.61%), with a broad-sense heritability of 0.93. The strong associations of DTF with genes that have orthologs with analogous functions in soybean and Arabidopsis provide strong circumstantial evidence that these genes are causal for this trait. The three reported loci and candidate genes provide useful targets for marker-assisted breeding in mung beans.

**Supplementary Information:**

The online version contains supplementary material available at 10.1186/s12864-024-10156-x.

## Introduction

Days to flowering (DTF), an important agronomic trait, marks the transition from vegetative to reproductive development. Flowering timing has a high positive correlation with crop maturity and hence yield [1]. As such, plants have evolved complex regulatory networks to ensure survival across various environmental conditions. Flowering time is regulated by environmental signals, such as temperature and day length, and endogenous pathways, including vernalization and the autonomous floral-promotion pathway [2, 3]. The Arabidopsis flowering pathway involves over 400 genes, some of which can be explored via the Flowering Interactive Database (FLOR-ID, http://www.flor-id.org/, accessed February 23, 2023) [4, 5] Notable among these genes are the *FLOWERING LOCUS C* (*FLC*), *FLOWERING LOCUS T* (*FT*), *FRIGIDA* (*FRI*), and *SUPPRESSOR OF OVER EXPRESSION OF CO1* (*SOC1*). *FLC* operates by suppressing the expression of *FT* and *SOC1,* which promote flowering [3, 5]. In soybean, classical loci with strong effects on DTF include *E1* through *E10* and the JUVENILE (*J*) locus [6–10]. For most of these, the orthologous counterparts have been identified in Arabidopsis [6, 7, 11].

Mung bean (*Vigna radiata* (L.) Wilczek), a warm-season pulse crop, is a valuable source of high-quality proteins, folates, carbohydrates, and vitamins. It contributes to a balanced diet by complementing cereals in South Asia, Africa, and South America [12, 13]. Originally domesticated in India, mung bean has successfully spread to various regions, including the northern and southern latitudes [14]. Selective breeding has resulted in lines adapted to the prolonged summer days and short nights of higher latitudes. In the Midwestern United States, there are several ongoing studies to support the introduction of mung bean as a cash crop through double or multiple cropping systems [12, 15–18]. Varieties exhibiting early flowering and maturation are preferred in such scenarios, making these important traits for mung bean improvement. The present work aims to facilitate selection for these traits through technologies such as marker-assisted breeding and genomic selection [19–23].

In this study, we report association mapping for DTF and evaluate shortlisted candidate genes relative to genes with established functions related to DTF in soybean and Arabidopsis.

## Materials and methods

### Phenotypic data collection

The Iowa Mung bean Diversity (IMD) panel reported by Sandhu and Singh [12] consists of 482 diverse accessions selected from the USDA germplasm collection (~ 3000 accessions) and commercial varieties from the World Vegetable Center (https://avrdc.org/). DTF data was collected in 2019 and 2021 near Ames, Iowa [12]. The panel was planted at the Iowa State University Agricultural Engineering and Agronomy (AEA) and Bruner farms near Boone, Iowa. A randomized complete block design (RCBD) was used with two replicates at each location. Each single-row plot was 7 ft. long, with 30 inches row-to-row spacing, and 50 seeds were planted per plot spaced 2 inches apart. The date when the first flower was observed in a plot was recorded, and the DTF was calculated from the planting date. This approach has been used in other species [24, 25] and is important here as the mung bean lines are not advanced and still show variation in DTF. We note that the panel was also planted in 2020, but the data from that year were compromised and discarded because of a derecho that swept across the Midwest on August 10. The derecho acted as a stressor leading to premature flower-drops, followed by a second flowering flush.

### Descriptive statistics

Analysis was conducted on 478 accessions planted in both 2019 and 2021. The mixed model below was used for the calculation of the best linear unbiased predictors (BLUPs) within the “inti” R package [26]:

$${Y}_{{\text{ijk}}} =\upmu + {(1|{\text{accession}})}_{i} + {{\text{loc}}\_{\text{year}}}_{j} + {(1|{\text{accession}}:{\text{loc}}\_{\text{year}})}_{ij} + {(1|{\text{loc}}\_{\text{year}}:{\text{bloc}})}_{k(j)} + {e}_{{\text{ijk}}}$$

where ${Y}_{{\text{ijk}}}$ is the phenotypic value (DTF) of the i^th^ genotype in the j^th^ environment (location*year combination) and k^th^ block, $\upmu$ is the overall mean, ${(1|{\text{accession}})}_{i}$ is the random effect due to the i^th^ genotype, ${{\text{loc}}\_{\text{year}}}_{j}$ is the fixed effect due to the j^th^ environment, ${(1|{\text{Accession}}:{\text{loc}}\_{\text{year}})}_{ij}$ is the random effect due to the i^th^ genotype * j^th^ environment interaction, ${(1|{\text{loc}}\_{\text{year}}:{\text{bloc}})}_{k(j)}$ is the random effect due to the k^th^ block nested within the j^th^ environment and ${e}_{{\text{ijk}}}$ is the random error following N(0, σe2). Broad-sense heritability was estimated as described by Cullies et al. [27]. Variance components and BLUPs were estimated. Analysis was conducted in the open-source R statistical computing environment [28].

### Single nucleotide polymorphism (SNP) coordinate update

The recently published mung bean reference genome version 7 for accession VC1973A [29] was used for positional analysis. To take advantage of this new genomic data, we projected the SNPs coordinates onto assembly version 7 from SNPs identified previously using genotyping-by-sequencing (GBS) [12] with coordinates relative to reference genome version 6 [30]. To project the sequences from assembly version 6 to version 7, the flanking 1001 bases around version 6 SNP coordinates were first extracted, (from 500 bases before the SNP to 500 bases after it). Retrieval of the flanking sequences was accomplished by deriving a four-column BED file with the molecule ID, the start and end coordinates, and the marker name. The sequence described by the BED file was extracted using the bedtools “getfasta” utility. The flanking sequences from the assembly version 6 were then used as queries for a blastn search, with parameters "-evalue 1e-10 -outfmt 7 -perc_identity 99". This tabular BLAST output was then filtered (using a simple awk script) to select the top match among those BLAST hits with 99% identity—with the further requirement that the match length be at least 990 bases. Of the initial 26,550 SNPs, 23,590 remained for downstream analysis after filtering out those with minor allele frequencies < 0.01 and > 15% missing data. Reference genome version 7, annotations, markers, and variant data can be found at the Legume Information System (LIS) Datastore (https://data.legumeinfo.org/Vigna/radiata/).

### Genome-wide association study (GWAS)

Genome-wide association mapping for DTF was conducted using both single loci mixed-linear model (MLM) [31] and multi-locus FarmCPU [32]. Both methods were implemented within the Genome Analysis and Prediction Integrated Tool (GAPIT) [33]. To control population structure, both the principal components (set PCA.total = 3) and the default kinship matrix were generated within GAPIT and used as covariates in the model. From the GWAS results, SNPs on chromosomes were visualized on Manhattan and Q-Q plots using CMplot [34] and qqman [35] packages. A significant threshold of -log_10_(1e-5) with a Bonferroni correction was used.

### Candidate gene identification

The two most statistically significant SNPs were identified for significant downstream analysis due to the fact their *p*-values were ~ 3 × those of the next four significant SNPs while accounting for ~ 65% of the phenotypic variance explained (PVE) by the significant SNPs (39.61%). We evaluated a 290 kb region on either side of each of the top two SNPs (580 kb total per locus) (Additional file 1 and 2 for scripts used and Additional file 3 and 4 for genes and fasta files). The 290 kb distance was selected as this is the average linkage disequilibrium (LD) decay within the IMD population [12]. A similar method had previously been applied in soybeans to find genes associated with iron deficiency chlorosis [36] as well as in mung bean to scan a 1 Mb region flanking soybean flowering genes [11].

BLASTP [37] was used to query the mung bean coding sequences identified above against all proteins in Arabidopsis (Araport11, [38], using an E-value cutoff of 1e-10. Matches were evaluated for functional descriptions associated with flowering time or maturity and publications in the TAIR database related to these traits. Additionally, the conserved domain search function at NCBI was used to identify domains present in Arabidopsis and mung bean proteins. Identified domains were visualized using PROSITE (https://prosite.expasy.org/) [39].

### Comparative genomics candidate gene identification

For comparative genomic analyses, we used the following tools available at the Legume Information System (LIS; legumeinfo.org) [40, 41]: ZZBrowse, Genome Context Viewer (GCV), JBrowse, Gene Family Search, Funnotate, and Phylogenetic tree viewers [42, 43]. The SoyBase (https://www.soybase.org/) “Convert Gene Model Names” tool [44, 45] was used to determine correspondences between different versions of soybean gene models.

### Gene family and phylogenetic analysis

Gene families for the *FERONIA*, *PhyA*, and *PIF3* families were calculated using the Pandagma workflow [46], on the set of all predicted protein sequences for the included species: *Vigna radiata* (VC1973 genome 7 annotation 1), *Vigna unguiculata* (IT97K-499–35 genome 1 annotation 2), *Glycine max* (Williams 82 genome 4 annotation 1), *Medicago truncatula* (A17_HM341 genome 4 annotation 1), *Lupinus albus* genome 1 annotation 1, *Cicer arietinum* (CDC Frontier genome 3 annotation 1), *Lotus japonicus* (MG20 genome 3 annotation 1), and *Arabidopsis thaliana* (Col0 genome 9 annotation 11). The source for all these annotations was legumeinfo.org (https://data.legumeinfo.org and https://data.legumeinfo.org/annex/; Redsun et al. 2022); metadata for each is in the respective collection folders in the annotation for each (Supplementary Table 1). The gene family construction approach uses MMSeqs2 [47] to identify pairwise matches between each protein set; then DAGChainer [48] to filter the matches by synteny. For those filtered matches, further filtering is done based on synonymous-site changes (Ks values) that are calculated in in-frame aligned coding sequence, using the PAML package [49]. The filtered results are then clustered with Markov clustering (mcl; [50] to generate provisional gene families. Sequences without family placements to this point are then compared against the provisional gene families and added to the top-matching family if homology thresholds are met (protein identity of at least 30% and alignment coverage of at least 40%). Protein sequences in each gene family were aligned using FAMSA [51]. From these alignments, hidden Markov models (HMMs) were calculated using the HMMER4 package [52]; hmmer.org). The family sequences were then realigned to the HMMs, and match-state characters were removed prior to calculating gene phylogenies. Finally, phylogenetic trees were calculated from cleaned alignments using FastTree v2.1 [53].

## Results

### Descriptive statistics and variance components

**Fig. 1:**
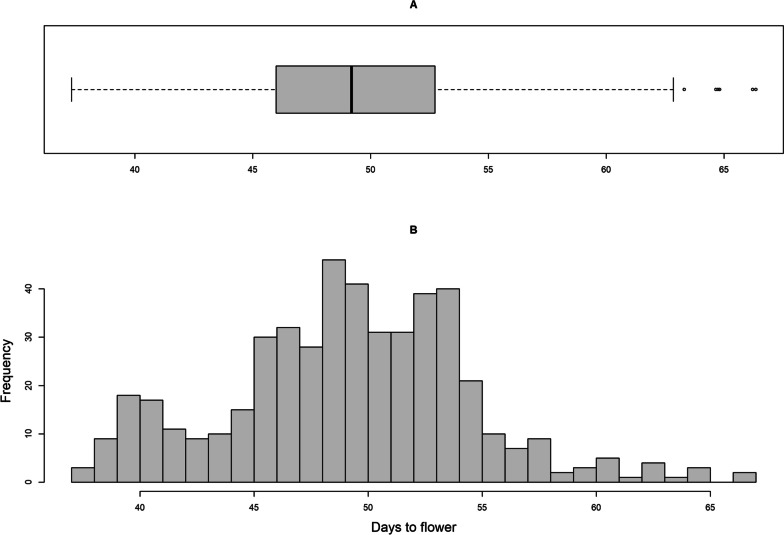
Boxplot (**A**) and histogram (**B**) illustrating the distribution of mung bean days to flowering (DTF) BLUPs

DTF in mung bean exhibited an approximately symmetrical distribution of the measured value, with a mean and median of 49 days, a standard deviation of 5 days, and a coefficient of variation of 10% (Fig. 1). As shown in Table 1, the accession effect accounted for the highest variation at 71.4%, followed by the effect of the interaction between accession and location-year at 13.8%, while the location-year/block interaction effect accounted for 1.2% (Table 1). DTF exhibited a high broad-sense heritability of 0.93.

**Table 1:** Variance components from the linear mixed model used in this study

Source	Variance	Percent Variation
Accession	30.93	71.4
Accession: location year	5.99	13.8
Location year: bloc	0.5	1.2
Residual	5.92	13.7
Totals	43.34	100.1

### SNP coordinate projection between assemblies

**Fig. 2:**
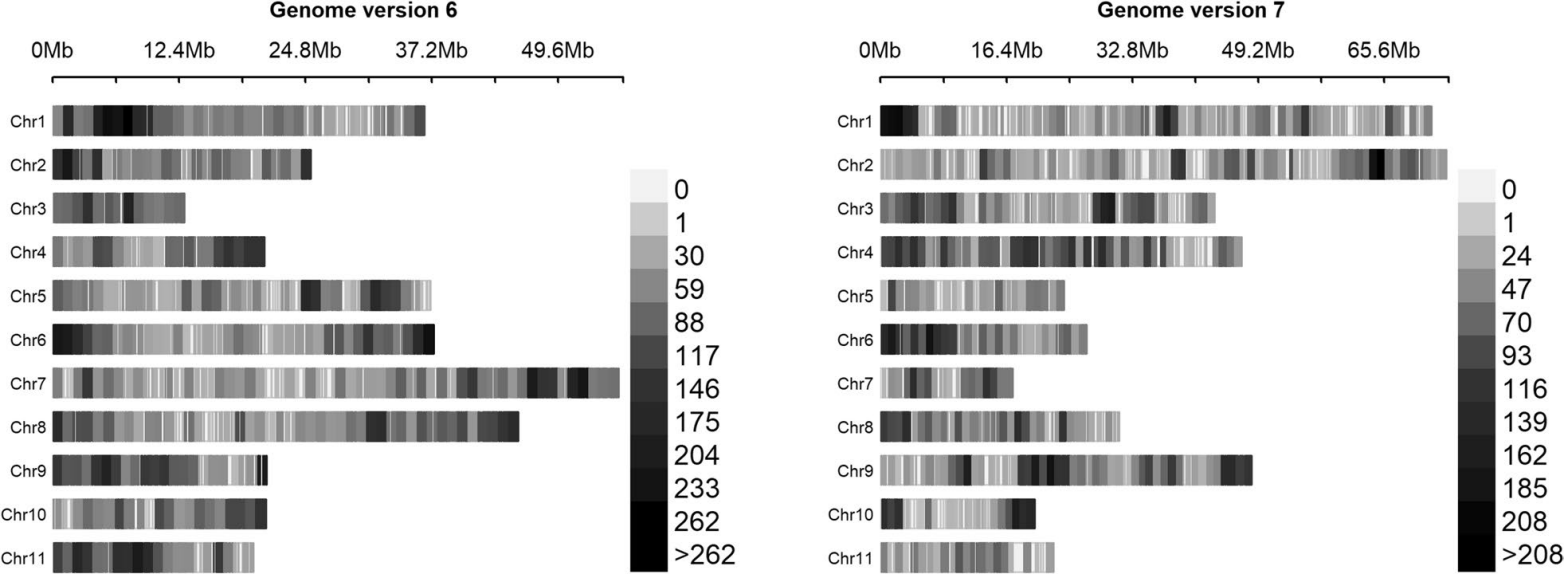
Iowa Mung bean Diversity panel SNP density plots after updating the SNP coordinates from the old genome version 6 (left) to the new genome version 7 (right) within a 1 Mb window size. X-axis shows the chromosome length, while the scale indicates SNP densities

Mapping the IMD panel SNPs from genome assembly version 6 to version 7 substantially rearranged the SNP locations, due to large structural changes between these two assemblies (Fig. 2). For example, from Fig. 2, it is apparent that the lengths of chromosomes 1, 2, 3, 4 and 9 increased in assembly 7, while those of chromosomes 5, 6, 7, 8, 10 and 11 decreased. Ha et al. [29] reported these rearrangements between these two assembly versions.

### GWAS results

**Fig. 3:**
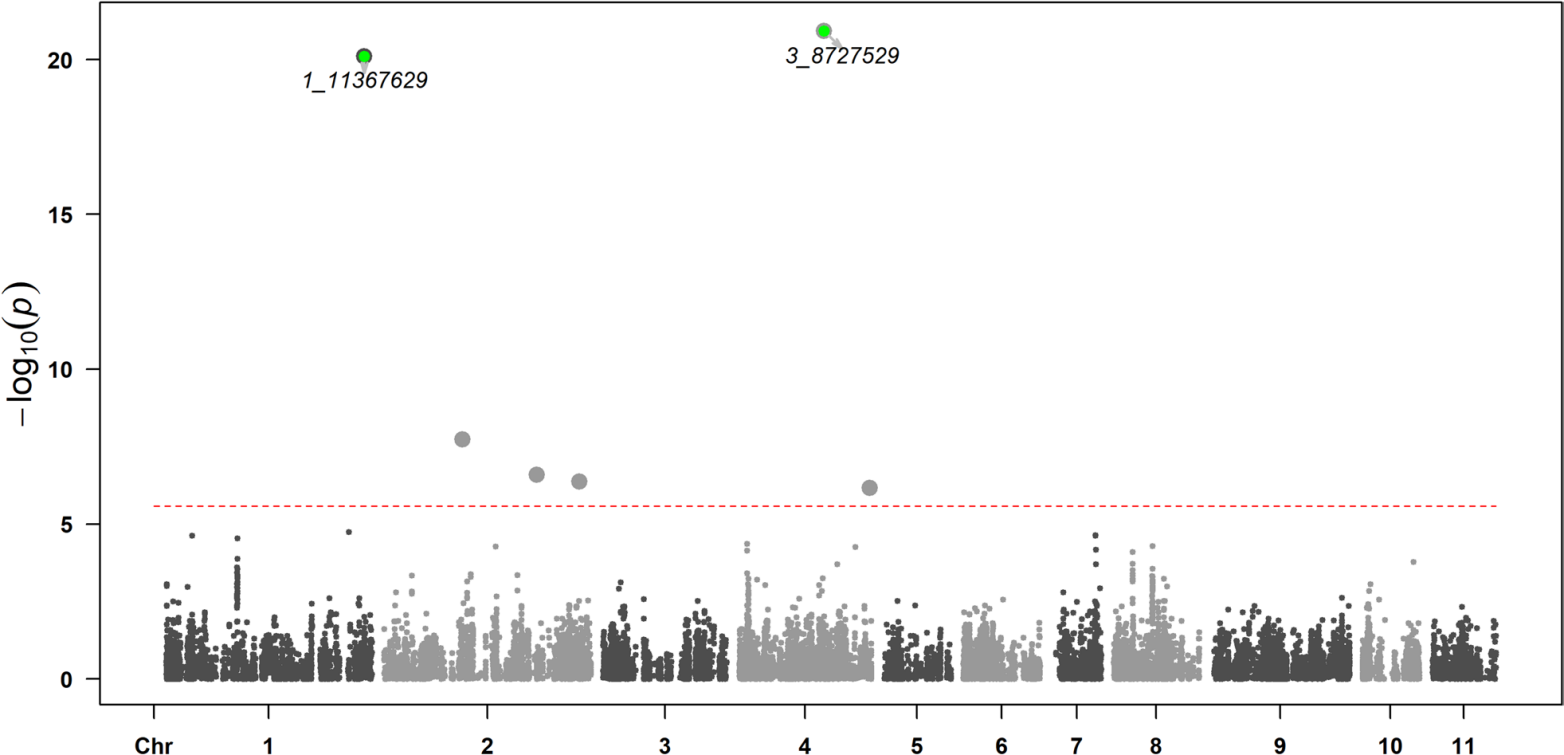
Manhattan plot showing significant SNPs associated with DTF. SNPs are labeled with the SNP name. SNPs in green are discussed further in the text. Trait-SNP associations were performed using FarmCPU in GAPIT. The horizontal dotted red line represents the Bonferroni correction as *p* = 0.05. See the accompanying Q-Q plot in Additional Fig. 1

MLM and FarmCPU methods resulted in similar significant SNPS; hence, only the results from FarmCPU are reported here due to the strength of multi-locus models [32]. Six SNPs associated with DTF were observed with *p*-values below 1e-5 (Fig. 3). The six SNPs were 1_11367629 (Chr 1), 8_3586220 (Chr 2), 5_4604047 (Chr 2), 5_430302 (Chr 2), 2_10755945 (Chr 4), and 3_8727529 (Chr 4) (Fig. 3 and Table 2). We note that the SNP names were assigned relative to assembly version 6 and should not be construed to have meaning relative to assembly 7. For example, SNP 1_11367629 remained on chromosome 1 in assembly 7, while SNPs 2_10755945 and 3_8727529 were relocated to chromosomes 4. As shown in Table 2, the SNPs 3_8727529 and 1_11367629 accounted for 13.72% and 11.84% of the phenotypic variance, respectively. In total, they accounted for 25.56% of the phenotypic variance explained (PVE) for DTF. The remaining four statistically significant SNPs only accounted for an additional 14.05% of the PVE and were thus not considered in the remaining analyses (Table 2). Our results validate previous reports of significant SNP associated with DTF in mung bean [12], with an additional year of data.

**Table 2:** The most significant SNPs from GWAS GAPIT results

SNP	Chromosome (chr)	Position (bp)	*P*-value	Phenotypic Variance Explained % (PVE)
3_8727529	4	29,758,658	1.21E-21	13.72
1_11367629	1	69,228,460	7.90E-21	11.84
8_3586220	2	53,453,008	2.55E-07	5.27
5_4604047	2	27,377,521	1.83E-08	4.05
5_430302	2	68,312,851	4.27E-07	3.52
2_10755945	4	45,689,185	6.75E-07	1.21
			**Total**	39.61

### Candidate gene evaluation

The 580 kb region associated with SNP 1_11367629 (on chr 1) contained 39 genes, while the 580 kb region associated with SNP 3_8727529 (on chr 4) contained 65 genes. For each of these 104 genes, the top Arabidopsis match was identified (https://arabidopsis.org/, accessed February 23, 2023) and checked for publications associated with flowering in Arabidopsis (Table 3). This search yielded 18 mung bean genes with such homologies (Table 3). Gene family and phylogenetic analyses were also conducted for the strongest candidate genes (Figs. 6 and 8).

**Table 3:** Mung bean genes orthologous to Arabidopsis genes involved in flowering. Genes within the 580 kb region surrounding the two most significant SNPs with homology (BLASTP, E-value 1e-10) to candidate genes from Araport11. Papers supporting the role of the identified protein in DTF processes are listed in the Supporting Paper column

SNP	Mung bean Gene ID	Araport11	E-value	% ID	Protein	Supporting Paper
	*Vradi01g00003484*	*AT3G51550*	1.74E-180	45.97	FERONIA	[54]
	*Vradi01g00003485*	*AT3G51550*	7.63E-14	60.00	FERONIA	[54]
	*Vradi01g00003486*	*AT3G51550*	1.61E-68	38.36	FERONIA	[54]
	*Vradi01g00003487*	*AT3G51550*	4.48E-88	51.17	FERONIA	[54]
	*Vradi01g00003488*	*AT3G51550*	0	46.40	FERONIA	[54]
	*Vradi01g00003489*	*AT3G51550*	0		FERONIA	[54]
	*Vradi01g00003490*	*AT3G51550*	3.05E-159	51.71	FERONIA	[54]
	*Vradi01g00003492*	*AT3G51550*	0	43.72	FERONIA	[54]
	*Vradi01g00003493*	*AT3G51550*	2.53E-47	41.26	FERONIA	[54]
	*Vradi01g00003494*	*AT3G51550*	3.79E-97	39.70	FERONIA	[54]
	*Vradi01g00003495*	*AT3G51550*	1.18E-120	57.84	FERONIA	[54]
	*Vradi01g00003497*	*AT3G51550*	6.75E-129	43.77	FERONIA	[54]
	*Vradi01g00003502*	*AT3G51550*	0	50.30	FERONIA	[54]
	*Vradi04g00002773*	*AT1G09570*	0	74.45	PhyA	[55]
	*Vradi04g00002778*	*AT1G09530*	1.12E-57	34.60	PIF3	[9]
	*Vradi04g00002804*	*AT1G61040*	2.21E-26	65.15	VIP5	[3]
	*Vradi04g00002805*	*AT1G61040*	7.46E-175	56.66	VIP5	[3]
	*Vradi04g00002812*	*AT1G23380*	4.31E-86	53.64	KNAT6	[56]

**Fig. 4:**
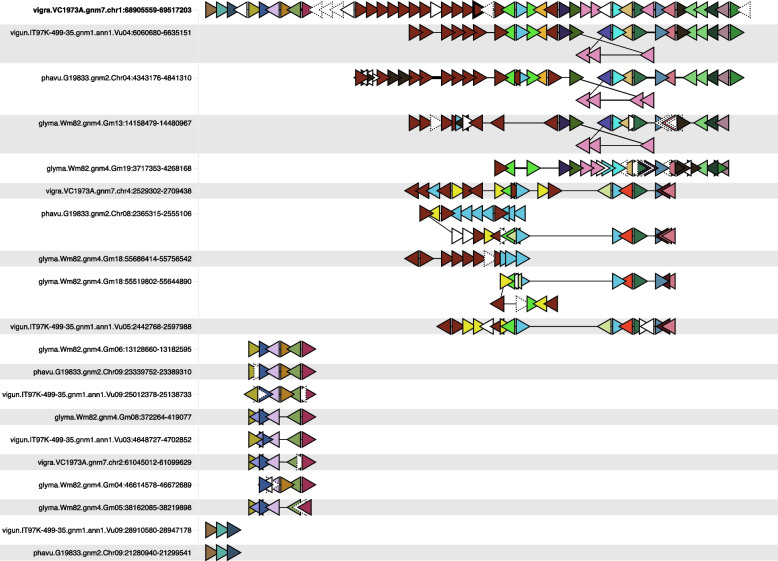
The region around SNP 1_11367629, showing *FERONIA* gene cluster. *FERONIA* genes are shown as brown triangles near the center. The gene containing the SNP with the strongest DTF association (*Vradi01g00003495*) is in the top row, center, with a black border. Genes from other gene families are indicated by other colors (one color per gene family). The triangle direction indicates gene orientation. Singleton genes are shown in white (dotted lines further indicating orphan genes, without gene family orthology). Crossed diagonal lines, for example, in the second row, indicate inversions. The text on the left indicates genus and species: vigra = *Vigna radiata*; vigun = *Vigna unguiculata*; phavu = *Phaseolus vulgaris*; glyma = *Glycine max*. Chromosomes and region coordinates are indicated on the left portion of the strings

**Fig. 5:**
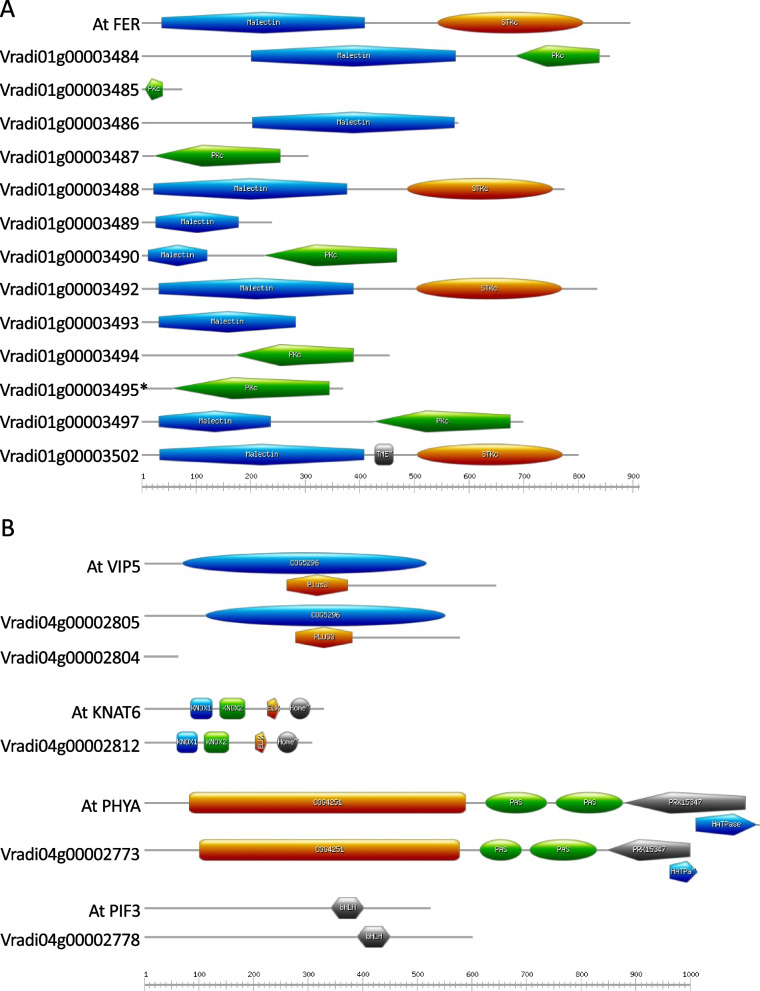
Domains of the primary candidate gene families: FERONIA, PhyA, and PIF3. **A**: Conserved domain analysis of Arabidopsis Feronia (*AtFER*) and 13 mung bean genes within the QTL that are identified as *FER* using homology and gene family analyses. Conserved domains are color and shape coded. The SNP was identified within the coding region of *Vradi01g00003495*, denoted with an *. This analysis supports *Vradi01g00003488*, *Vradi01g00003492*, and possibly *Vradi01g00003502* as the most likely candidate genes. **B**. Conserved domain analysis of Arabidopsis candidate genes and the mung bean homologs. Note, no conserved domains were identified in the *Vradi04g00002804* sequence. Other than that gene, all domains identified in Arabidopsis, were identified in the mung bean homologs

**Fig. 6:**
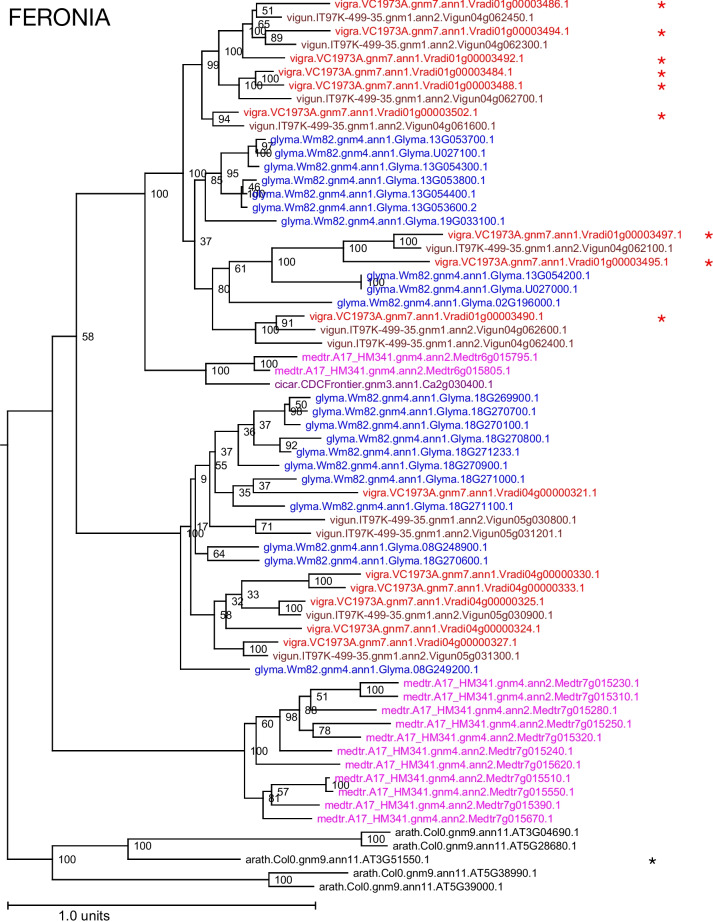
*FERONIA* gene family for selected legume species. *Arabidopsis* orthologs are shown in gray at the bottom, including the classical *FER* gene (*AT3G51550*), marked with a black asterisk. Each species is represented by a color and is indicated by the leading five-letter prefix in the gene name: arath = *Arabidopsis thaliana*; cicar = *Cicer arietinum*; glyma = *Glycine max*; medtr = *Medicago truncatula*; vigun = *Vigna unguiculata*; vigra = *Vigna radiata*; lupal = *Lupinus albus*. The *V. radiata* paralogs in the cluster on chromosome 1 are marked with red asterisks

For SNP 1_11367629 on chromosome 1, the SNP occurs within a cluster of 13 orthologs of the Arabidopsis gene *FERONIA* (*FER*) (Fig. 4). These *FER* orthologs were *Vradi01g00003484*-*Vradi01g00003490* (seven genes), *Vradi01g00003492*-*Vradi01g00003495* (4 genes), *Vradi01g00003497,* and *Vradi01g00003502*. The SNP occurs within the single exon of *FER* gene *Vradi01g00003495.* The C/T allele in *Vradi01g00003495* is a missense mutation, with the observed C and T variants coding for histidine 82 (CAC) and tyrosine (TAC), respectively. The conserved domain analyses revealed that only proteins encoded by the genes *Vradi01g00003488*, *Vradi01g00003492*, and *Vradi01g00003502* share the same two conserved domains with the Arabidopsis FER protein (AT3G51550) (Fig. 5). However, gene family and phylogenetic analyses (Fig. 6), based primarily on sequence from the Malectin domain (IPR024788), show the *FER* gene family to be large and complex, with expansions and contractions of clusters – as evident in the cluster of *FER* homologs on mung bean chromosome 1. Similarly, in Arabidopsis, there are five *FER* paralogs in two clusters – one on chromosome 3 and one on chromosome 5. From the perspective of that domain, all 15 *FER* genes in *V. radiata* are orthologous to the five *FER* genes in *A. thaliana*, likely deriving from a common shared ancestral gene.

**Fig. 7:**
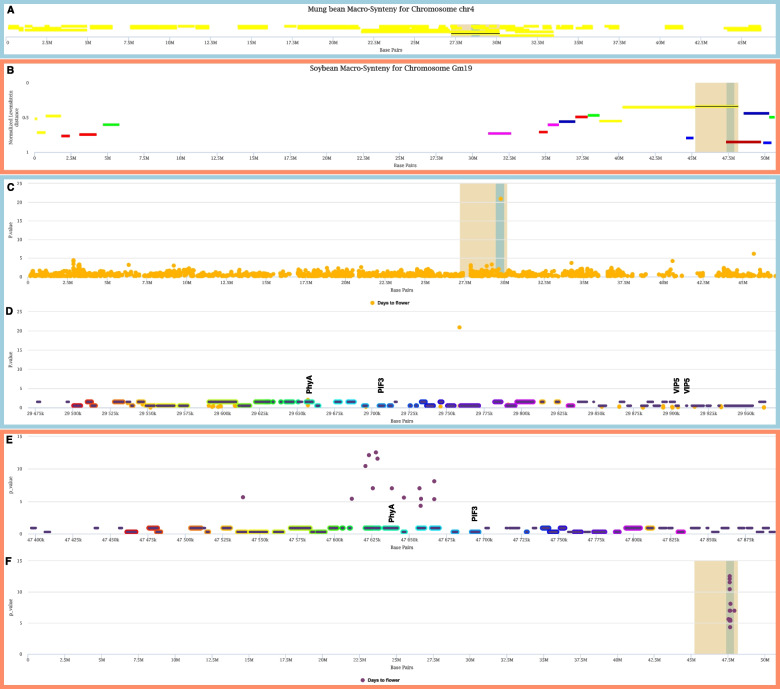
Comparison of syntenic regions for Days to Flowering loci in mung bean and soybean. **A**. Mung bean chromosome 4. Regions in synteny analysis are in yellow. A light blue slice near 30 Mb is highlighted in synteny search in soybean in next panel. **B**. Soybean chromosome 19. Colored horizontal regions indicate synteny with mung bean chromosomes. Light blue slice near 47 Mb is orthologous to the slice highlighted in A. **C**. Mung bean markers from GWAS. A blue slice around significant SNP near 30 Mb is shown in the inset in the next panel. **D**. Zoomed region of 250 kb corresponding with a slice in C. Gene models are shown at the bottom of panel. Rainbow colors show orthology with soybean genes in the next panel. Genes with annotations related to flowering are highlighted above each gene: PhyA, PIF3, VIP5. **E**. Soybean region from chromosome 19 corresponds via synteny with regions above from mung bean chromosome 4. The figure is adapted from ZZBrowse [41]

**Fig. 8:**
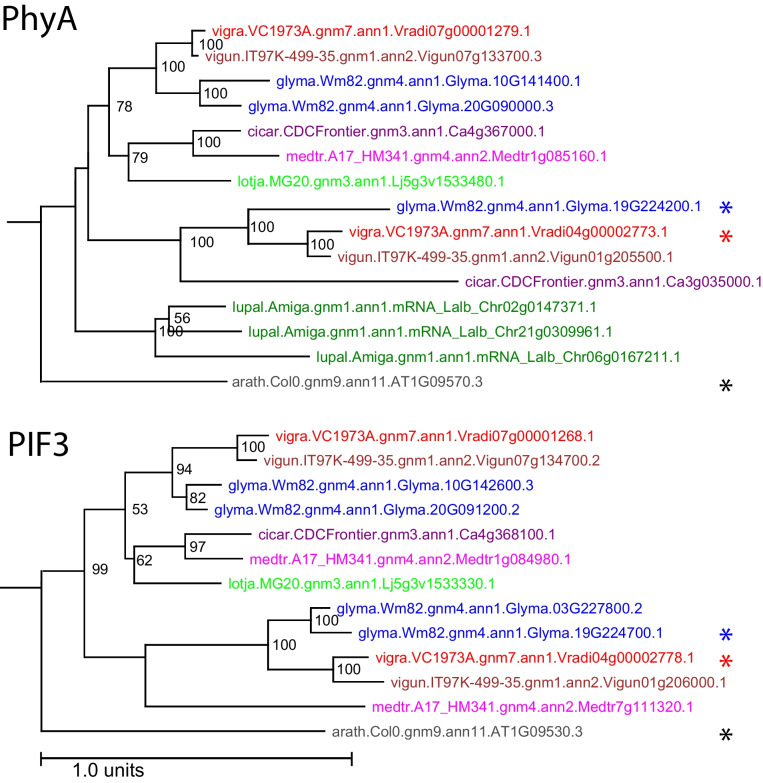
*PhyA* and *PIF3* gene families for selected legume species. *Arabidopsis* orthologs are shown in gray at the bottom, including the classical genes marked with a black asterisk: *PhyA*, (*AT1G09570*; top); and *PIF3* (*AT1G09530*; bottom). Orthologs in *V. radiata* closely linked to the DTF trait are marked with a red asterisk. Each species is represented by a color and is indicated by the leading five-letter prefix in the gene name: arath = *Arabidopsis thaliana*; cicar = *Cicer arietinum*; glyma = *Glycine max*; lotja = *Lotus japonicus*; medtr = *Medicago truncatula*; vigun = *Vigna unguiculata*; vigra = *Vigna radiata*; lupal = *Lupinus albus*

**Fig. 9:**
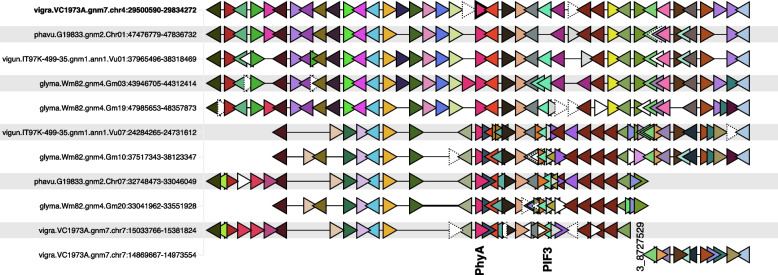
The region around SNP 3_8727529, showing candidate genes *PhyA* and *PIF3* and surrounding genes. The *PhyA* orthologs are shown in pink and *PIF3* in turquoise and labeled at the bottom (along with the approximate location of the DTF-associated SNP). The mung bean *PhyA* gene (*Vradi04g00002773*) is in the top row, center, with a black border. Genes from other gene families are indicated by other colors (one color per gene family). The triangle direction indicates gene orientation. Singleton genes are shown in white (dotted lines further indicating orphan genes, without gene family orthology). The text on the left indicates genus and species: vigra = *Vigna radiata;* vigun = *Vigna unguiculata*; phavu = *Phaseolus vulgaris*; glyma = *Glycine max*. Chromosomes and region coordinates are indicated on the left portion of the strings

For SNP 3_8727529 on chromosome 4, several genes in the 580 kb flanking region have orthologs with roles related to flowering in Arabidopsis, including *Vradi04g00002773*, *Vradi04g00002778*, *Vradi04g00002804*, *Vradi04g00002805*, *Vradi04g00002812* (Fig. 7)*.* These correspond to Arabidopsis genes phytochrome A (*PhyA*), phytochrome-interacting factor 3 (*PIF3*), vernalization independence 5 (*VIP5*, two copies), and knotted1-like from *Arabidopsis thaliana* 6 (*KNAT6*), respectively. The conserved domain analysis confirmed the mung bean genes perfectly reflected the domains in the Arabidopsis homologs (Fig. 5). Based on proximity to the DTF-associated marker on chromosome 4 and functional studies in Arabidopsis, both *PhyA* (*Vradi04g00002773*) and *PIF3* (*Vradi04g00002778*) are strong candidates for the causal genes. The gene family phylogenies for *PhyA* and *PIF3* are shown in Fig. 8, and synteny among legume genomes in the vicinity of chromosome 4 *PhyA* are shown in Fig. 9. The mung bean *PhyA* and *PIF3* orthologs are located at 29.66 and 29.70 Mb relative to SNP 3_8727529 at 29.75 Mb, in the 71.9 Mb chromosome. While the two *VIP5* genes and *KNAT6* are further from the SNP at 29.90 Mb (both *VIP5* genes) and 29.97 Mb relative to chromosome 4, they may play a role in mung bean DTF and contribute to the significance of SNP 3_8727529. In contrast, the ortholog to the gene *GmDt1*/ *GmTFL1b* (*Vradi04g00002442* in mung bean, *Glyma.19G194300* in soybean), associated with determinate growth in soybean [7], is on the same chromosome as the mung bean chromosome 4 DTF locus but is separated from it by 2.5 Mb, so we consider this an unlikely candidate.

## Discussion

Mung bean shares many of the nutritional and agronomic advantages of other legume crops and is relatively drought- and heat-tolerant, making it an increasingly important crop in the context of climate change [57–59]. Interest in mung bean can also be attributed partly to changing consumer habits in the West, associated with reducing meat as a primary protein source [60, 61].

The high heritability score (0.93) of DTF in mung bean makes it a good candidate for genomic-assisted breeding. Combining GWAS results with comparative methods, we have identified strong DTF candidate genes at two loci, orthologous to genes associated with DTF from soybean and Arabidopsis (Table 3).

The previous reference mung bean genome version 6 needed to be structurally corrected, as reported by Ha et al. [29]. Previous work done by our group identified two of the same SNPs (1_11367629 and 3_8727529) associated with DTF in mung bean [12], but errors in version 6 confounded marker-trait associations. The SNP coordinates have been updated in the genome version 7 sequence, with SNP 3_8727529 now located on chromosome 4.

For the chromosome 1 DTF locus associated with SNP 1_11367629, we identify the cluster of 13 mung bean homologs of the Arabidopsis receptor-like protein kinase *FERONIA* (*FER*) gene (*AT3G51550.1*), flanking SNP 1_11367629, as the best candidates. In version 7 of the genome SNP 1_11367629 is located within the single exon of *Vradi01g00003495*. It is intriguing that the C/T allele in *Vradi01g00003495* is a missense mutation (histidine-to-tyrosine); however, while it is possible that this mutation is responsible for a changed phenotype, it is also likely that there are structural or copy-number variations at this complex locus. Domain analysis of the 13 *FER* genes identified in mung bean and the *AtFER* reveal only three of the mung bean genes (*Vradi01g00003488, Vradi01g00003492* and *Vradi00003502*, but not *Vradi01g00003495*) share the same two conserved protein domains identified in Arabidopsis (Fig. 5). It is notable that *Vradi01g0003502* encodes a third domain, a transmembrane epidermal growth factor receptor-like domain. The two conserved domains are a Malectin domain and a serine/threonine kinase catalytic (STKc) domain. Two of the mung bean *FER* genes encode a protein kinase catalytic (PKc) domain instead of the serine/threonine kinase catalytic (STKc) domain present in Arabidopsis. Both are protein kinase domains, but the STKc domain specifically phosphorylates the hydroxyl (OH) group of serine or threonine while the PKc domain is not restricted to specific amino acids. The remaining seven genes only encode either the Malectin domain or the PKc domain. *FER* has been associated with various aspects of plant growth, including flowering, root hair development, and hypocotyl and root elongation. In Arabidopsis, *FER* has been shown to regulate flowering time by up-regulating the expression of *FLOWERING LOCUS C* (*FLC*) [12, 54, 62]. Since *FER* exists as a cluster of locally duplicated genes, we speculate that copy number variation in this locus may affect the maturity continuum in mung beans as reflected by the days to flowering. Given the prevalence of the Malectin and PKc domain encoding proteins in this region, we hypothesize these genes may not be major DTF genes like *FER* but may serve to micro-regulate DTF.

The *FERONIA* gene phylogeny (Fig. 6) and synteny depiction (Fig. 4) both bear indications of actively evolving clusters in the included species. Genes in the *V. radiata* clusters on chromosomes 4 and 1 are interleaved with syntenic clusterson *V. unguiculata* chromosomes 4 and 5 respectively, and *G. max* chromosomes 13, 2, 18, and 8. The interleaving in the phylogeny implies that the clusters on *V. radiata* chromosomes 1 and 4 are at least as old as their common ancestor (the warm-season legume ancestor). On the other hand, one of the two post-papilionoid WGD clusters in *Medicago* has expanded while the other has not.

Finally, for SNP 3_8727529 on chromosome 4, there were several orthologs to Arabidopsis genes with roles associated with the control of flowering, including *PIF3* (phytochrome interacting factor 3), *VIP5* (vernalization independence 5), *KNAT6*, which influences inflorescence architecture, and *PhyA* (phytochrome A; *AT1G09570*). Of these, we consider the strongest candidate to be the *Vigna PhyA* ortholog (*Vradi04g00002773*) due to its proximity to the SNP (98.5 kb) and the strong syntenic relationship with the soybean orthologs, *E3/GmPhyA3* and *E4/GmPhyA2*. In soybean, the paralogs *E3* and *E4* (derived from the *Glycine* whole-genome duplication) suppress flowering but do so in response to varying light qualities [55]. In soybean, *E3* shows the strongest GWAS signal for DTF in soybean (Kim et al., 2020) (Fig. 8). This gene also aligns with the conclusions of Hwang et al. [63], and Ha et al., [29]. Both of these publications identified *Vradi04g00002773* as the best candidate for several mung bean QTLs including flower initiation (F14-1), number of nodes (Node4-1), synchronous pod maturity (SPM4-1) and plant height [29]. These findings support our previous findings performed in the United States and those from South Korea. Despite the importance of *PhyA*, we would be remiss not to consider the other candidate genes located within this genomic region. While *PhyA* may be the major gene associated with SNP 3_8727529, the other genes associated with DTF in this genomic region may also play supporting roles in regulating mung bean DTF. In Arabidopsis, silencing *PIF3* results in early flowering by increasing the levels of *FT* [64]. Similarly, silencing any of the *VIP* genes causes silencing of *FLC*, resulting in early flowering [3, 65]. Given the role of these genes in Arabidopsis, it’s plausible that they also contribute to the DTF phenotype measured in this study, despite being further from the SNP of interest. Previous studies in soybean and other species have confirmed that multiple genes may be involved in conferring a QTL, suggesting the same would be true for mung bean.

### Supplementary Information

**Supplementary Material 1**.

**Supplementary Material 2**.

**Supplementary Material 3**.

**Supplementary Material 4**.

**Supplementary Material 5**.

**Supplementary Material 6**.

**Supplementary Material 7**.

## Data Availability

The dataset(s) supporting the conclusions of this article is (are) included within the article (and its additional file(s).
