# Supplementary material for: Combining GWAS and comparative genomics to fine map candidate genes for days to flowering in mung bean
Source: BMC Genomics. 2024 Mar 12;25:270. doi: 10.1186/s12864-024-10156-x (PMC10935824; doi:10.1186/s12864-024-10156-x)
Supplement: Supplementary file 6 — Supplementary Material 6. [file 12864_2024_10156_MOESM6_ESM.pdf]

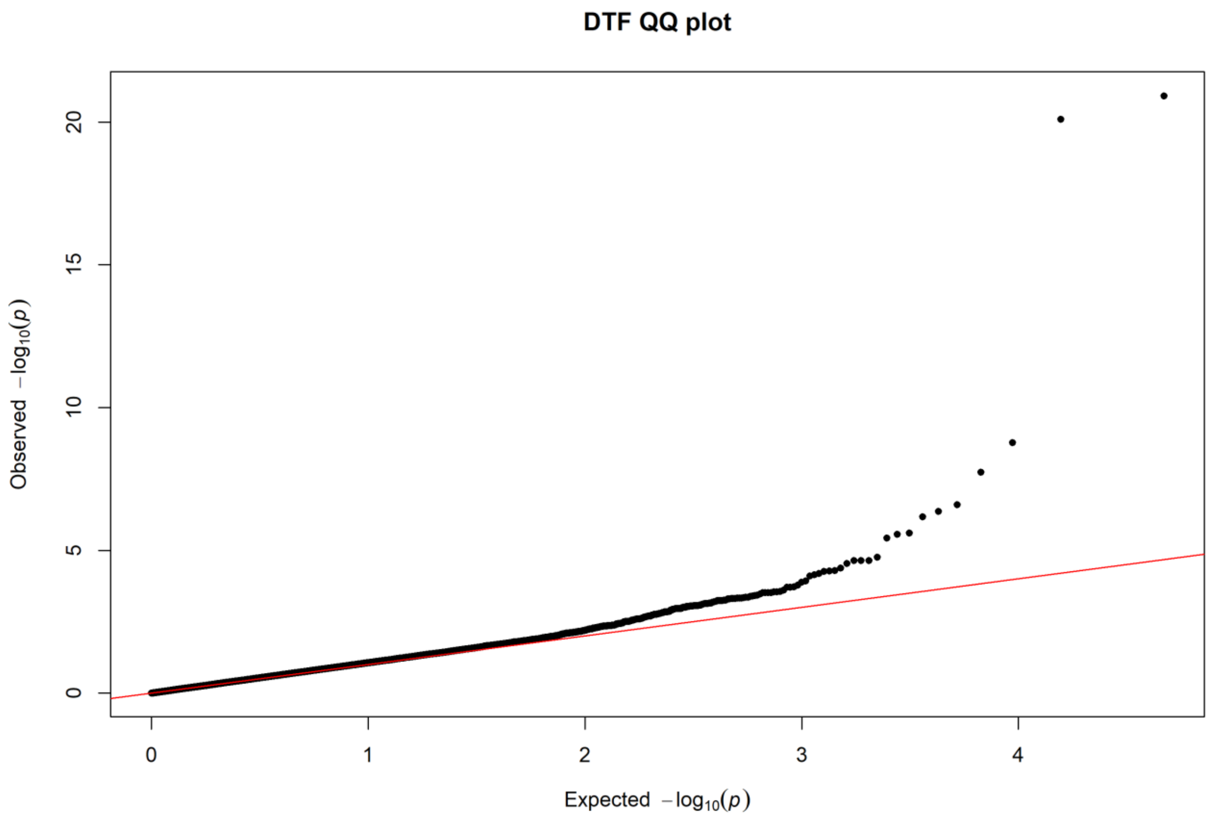

Additional Figure 1. Normal Quantile-Quantile Plot for DTF. The plot compares ordered value of DTF with quantiles of the gaussian distribution. If the data distribution matches the theoretical distribution, a linear pattern is observed.
