# Supplementary material for: Combining GWAS and comparative genomics to fine map candidate genes for days to flowering in mung bean
Source: BMC Genomics. 2024 Mar 12;25:270. doi: 10.1186/s12864-024-10156-x (PMC10935824; doi:10.1186/s12864-024-10156-x)
Supplement: Supplementary file 7 — Supplementary Material 7. [file 12864_2024_10156_MOESM7_ESM.docx]

**Supplementary Table 1: Metadata for each of the genomes used in gene and phylogenetic analysis.**

| *Vigna radiata* | <https://data.legumeinfo.org/Vigna/radiata/annotations/VC1973A.gnm7.ann1.RWBG/> |
| --- | --- |
| *Vigna unguiculata* | <https://data.legumeinfo.org/Vigna/unguiculata/annotations/IT97K-499-35.gnm1.ann2.FD7K/> |
| *Glycine max* | <https://data.legumeinfo.org/Glycine/max/annotations/Wm82.gnm4.ann1.T8TQ/> |
| *Medicago truncatula* | <https://data.legumeinfo.org/Medicago/truncatula/annotations/A17_HM341.gnm4.ann2.G3ZY/> |
| *Lupinus albus* | <https://data.legumeinfo.org/Lupinus/albus/annotations/Amiga.gnm1.ann1.3GKS/> |
| *Cicer arietinum* | <https://data.legumeinfo.org/Cicer/arietinum/annotations/CDCFrontier.gnm3.ann1.NPD7/> |
| *Lotus japonicus* | <https://data.legumeinfo.org/Lotus/japonicus/annotations/MG20.gnm3.ann1.WF9B/> |
| *Arabidopsis thaliana* | <https://data.legumeinfo.org/annex/Arabidopsis/thaliana/annotations/Col0.gnm9.ann11.KH24/> |
